# Effect of free and bound polyphenols from *Rosa roxburghii* Tratt distiller's grains on moderating fecal microbiota

**DOI:** 10.1016/j.fochx.2023.100747

**Published:** 2023-06-12

**Authors:** Die Zhou, Jiang Zhong, Yongguang Huang, Yuxin Cheng

**Affiliations:** a College of Liquor and Food Engineering, Key Laboratory of Fermentation Engineering and Biological Pharmacy of Guizhou Province, Guizhou University, Guiyang, Guizhou 550025, China; b Key Laboratory of Fermentation Engineering and Biological Pharmacy of Guizhou Province, Guiyang, Guizhou 550025, China

**Keywords:** *Rosa roxburghii* Tratt distiller’s grains, Polyphenols, Antioxidant activity, Fecal microbiota, Short chain fatty acids

## Abstract

*Rosa roxburghii* Tratt distiller's grains (*R. roxburghii* DGs), the main by-product of wine processing, showed functional value and potential for high-value usage which benefited from their rich polyphenols. In this study, the free and bound polyphenols from *R. roxburghii* DGs were extracted and their potential effect on modulating fecal microbiota was investigated using *in vitro* fecal fermentation. The free polyphenols (26.32–26.45 mg GAE/g) showed higher antioxidant activity compared to the bound polyphenols (8.76–9.01 mg GAE/g). The free and bound polyphenols significantly improved the fecal microbiota community structure and enhanced short chain fatty acids concentrations after the stimulated colonic fermentation for 24 h. Furthermore, the effect of *R. roxburghii* DGs polyphenols on modulating fecal microbiota was primarily attributed to quercetin, catechin, kaempferol, cyanidin and baicalin. This research suggests that *R. roxburghii* DGs are a promising source of natural antioxidants and prebiotic foods.

## Graphical abstract

**Figure:**
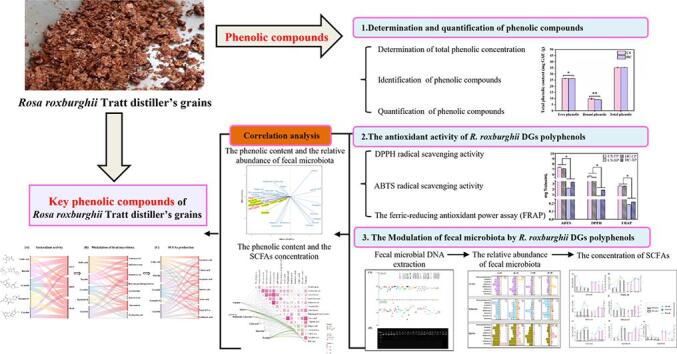

## Introduction

The gut microbiota, the whole of the intestinal microorganisms, had a ten times greater number than cells in gut. Many studies have suggested that the gut microbiota plays crucial roles in promoting host metabolic health, maintaining host homeostasis, and strengthening host immunity (Fan and Pedersen, 2021, Kamada et al., 2013, Lee et al., 2018). While, the host gut microbiota was vulnerable to negative alterations brought on by aging, diet and antibiotic usage (Fan and Pedersen, 2021, Kamada et al., 2013). This could result in aberrant microbial colonization in the gut and many prevalent metabolic problems, including type 2 diabetes, obesity and other diseases (Fan & Pedersen, 2021). Hence, it becomes essential to control gut microbiota homeostasis in order to maintain host health. Currently, sufficient evidences showed that diet and food components such as polyphenols can alter the gut microbial composition and promote intestinal health (Luo et al., 2019, Su et al., 2022, Wang et al., 2022, Wang et al., 2022).

Polyphenols, mainly flavonoids, tannins and phenolic acids, are natural antioxidants found in fruits and vegetables (Mithul Aravind et al., 2021). The significant antioxidant properties of phenolic compounds could scavenge free radicals, reducing intestinal oxidative stress levels and maintaining intestinal microecological balance status (Brezoiu et al., 2019). While due to their low bioavailability, only a small portion of free polyphenols were directly absorbed. The majority polyphenols were mainly collected in large intestine, where they showed influence on the composition of the intestinal microbiota by increasing or suppressing beneficial microbiota or bacteria with conditional pathogenicity (Chan et al., 2023, Cladis et al., 2021, Su et al., 2022). The supplementation with polyphenols from *Lycium ruthenicum* enhanced the growth of *Lactobacillus*, *Bifidobacterium* and *Akkermansia* and decreased *Prevotellaceae*, thereby regulating the immune system and maintaining host health (Luo et al., 2019). Meanwhile, their metabolites, like SCFAs, maintained intestinal microecological balance, provided energy and protected the integrity of the intestinal mucosa to preserve the host gut health (Koh et al., 2016). As a result, polyphenols were also regarded as an excellent source of prebiotics with great potential to modulate the gut microbes.

*R. roxburghii* fruit is one of the most distinctive resources in China, with Guizhou having the highest planted area and processing yield. This fruit rich in a variety of bioactive substances, especially flavonoids, tannins and phenolic acids (Li et al., 2022a). Due to its sour and astringent qualities, *R. roxburghii* fruit was always processed into *R. roxburghii* wine which showed an emerging high-economic-effect, currently. While *R. roxburghii* DGs, a by-product of *R. roxburghii* wine, were still abundant in bioactive substances and consistently neglected, resulting in a huge waste of resources and delaying the development of the wine industry and circular economy (Jiang et al., 2022, Li et al., 2022a). Previous studies have mainly examined the composition and functional activity of polyphenols from *R. roxburghii* fruit (Jiang et al., 2022, Li et al., 2022a, Li et al., 2022b, Liu et al., 2016) and pomace (Huang et al., 2022, Su et al., 2022). Research proved that the total polyphenols and flavonoids in *R. roxburghii* contributed more than 50% to the antioxidant activity, highlighting their significance in the antioxidation of *R. roxburghii* (Zhou et al., 2017). In addition, polyphenols from *R. roxburghii* fruit improved the intestinal microbiota in animals, increasing the population of beneficial bacteria while decreasing the abundance of harmful bacteria, according to recent research (Wang et al., 2022, Wang et al., 2022). These results suggest that *R. roxburghii* DGs which still rich in polyphenols may have important health benefits. Similarly, wine lees have been demonstrated to promote the growth of intestinal microbiota, which may be related to their abundance of polyphenols and dietary fiber (Gil-Sanchez et al., 2017). Furthermore, previous research has shown that phenolic compounds as well as dietary fiber demonstrate a synergistic health effect, enhancing the growth of beneficial microbiota (Su et al., 2022, Xu et al., 2019). These evidences will provide insight into the reuse of *R. roxburghii* DGs.

Therefore, in this study, the free and bound polyphenols from *R. roxburghii* DGs were extracted and their potential effect on modulating fecal microbiota was investigated using *in vitro* fecal fermentation. Besides, the important phenolic compounds from the polyphenols in *R. roxburghii* DGs that regulate intestinal health have been identified in this study. Furthermore, the health effects underlying the modulation of *R. roxburghii* DGs polyphenols on the fecal microbiota were deeply and comprehensively explained. Overall, this research offered a theoretical foundation for the high-value utilization of *R. roxburghii* DGs and their development as functional food ingredients.

## 2 Materials and methods

### 2.1 Materials and chemicals

Two kinds of *R. roxburghii* DGs named CS and HC were provided by Changshun Dnansoya *Rosa roxburghii* Farm Co. Ltd. (Guizhou, China) and Guizhou Hongcai Investment Group Co. Ltd. (Guizhou, China), respectively. *R. roxburghii* DGs were dried at 50 °C for 24 h until the moisture contents reached 6.6%–11.2%. The sample was crushed through the 40-mesh sieve and then stored at 4 °C for further experiment. The elementary components of *R. roxburghii* DGs are listed in Supplementary Table S1. Folin-Ciocalteau reagent was purchased from Solarbio Science & Technology Co. Ltd. (St Louis, MO, USA). 1-Diphenyl-2-picrylhydrazyl (DPPH), 2,2′-amino-di (2-ethyl-benzothiazoline sulphonic acid-6), ammonium salt (ABTS), 2,4,6-tris(2-pyridyl)-s-triazine (TPTZ), 6-hydroxy-2,5,7,8-tetramethylchroman-2-carboxylic acid (Trolox) and reference standards were obtained from Shanghai Maclin Biochemical Technology Co. Ltd. (Shanghai, China). Pepsin (porcine stomach mucosa, S10027, 1:30000 USP specifications) and pancreatin (S10031, 1:4000 BR specifications) was purchased from Yuanye Biotech Co. Ltd. (Shanghai, China). Bile pig powder (S30895, BR specifications) were purchased from Sigma-Aldrich Chemical Co. (St Louis, MO, USA). Other solvents used for HPLC analysis were chromatographic-grade (≥99.999%). The internal standard (2-ethylbutyric acid, ≥99.999%) and external standards (acetic, propionic, butyric, valeric, isobutyric and isovaleric acids, ≥99.999%) were purchased from Maclin Biochemical Technology Co. Ltd. (Shanghai, China) to determine the concentration.

### 2.2 Extraction of free and bound polyphenols from *R. roxburghii* DGs

The free polyphenols from *R. roxburghii* DGs were extracted by the solvent extraction method with some modifications (Yang et al., 2022). Firstly, the *R. roxburghii* DGs powder (2.0 g) was mixed with 20 mL of chilled 80% methanol (1% v/v formic acid). The mixture was sonicated for 30 min at 25 ℃ in the dark and then centrifuged (4,500 rpm, 10 min, 4 °C) to collect the supernatant (re-extracted twice). Then, the residue was mixed with 40 mL of chilled 80% methanol (1% v/v formic acid) and re-extracted twice with the same process. The combined supernatants were evaporated using a rotary vacuum evaporator (50 rpm, 30 min, 35 ℃) and then defatted with petroleum ether at a ratio of 1:2 (v/v) to obtain the free polyphenols.

The bound polyphenols from *R. roxburghii* DGs was extracted using the alkaline hydrolysis method reported previously (Su et al., 2022). The residue from the extraction of free polyphenols was mixed with NaOH (2 mol/L) at a ratio of 1:40 g/mL and stirred for 4 h under an oxygen free atmosphere. Afterwards, the pH value of the mixture solution was adjusted to 2.0 ± 0.2 by adding HCl solution (6 mol/L). The mixture was extracted with ethyl acetate at a 1:40 (m/v) and the organic fractions were collected by centrifugation at 8,000 rpm, 4 °C for 10 min. Subsequently, the combined organic fractions were evaporated using a rotary evaporator (50 rpm, 35 ℃) and defatted with petroleum ether at a ratio of 1:2 (v/v) to obtain the bound polyphenols.

### 2.3 Determination of total phenolic contents

The total phenolic contents, free and bound polyphenols, of *R. roxburghii* DGs was determined by the Folin-Ciocalteu method. Folin-Ciocalteu reagent (1 mL) and distilled water (5 mL) were added to 1 mL extract solution (1:50 and 1:10 diluted with distilled water, respectively). The Na_2_CO_3_ (3 mL, 7.5%) was added and mixed for 1 min. The mixture was incubated for 2 h at 25 °C and the absorbance was noted at 760 nm. The total phenolic contents were expressed as mg of gallic acid equivalents (GAE) per gram of a sample of *R. roxburghii* DGs (mg GAE/g).

### 2.4 Determination and quantification of free and bound polyphenols from *R. roxburghii* DGs

The compositions of free and bound polyphenols from *R. roxburghii* DGs were analyzed using an ultraperformance liquid chromatography system (Shim-pack UFLC SHIMADZU CBM A system) connected to a Q-Exactive (Thermofisher Scientific, USA) equipped with an ESI source spectrometer (UPLC-ESI/QE-MS/MS). UPLC separations was carried out with an ACQUITY UPLC HSS T3 C18 column (100 × 2.1 mm, 1.8 µm) (Waters, Milford, MA, USA). The column maintained at 40 ℃. The flow rate and injection volume were set at 0.4 mL/min and 2 µL, respectively. The mobile phases consisted of (B2) 0.1% formic acid in acetonitrile (v/v) and (A2) 0.1% formic acid in water (v/v). Separation was conducted under the following gradient: 0 min, 5% B; 10 min, 95% B; 11 min, 95% B; 11.1 min, 95% B; 15 min, 95% B. MS experiments work were as follows: sheath gas pressure, 30 arb; aux gas flow, 10 arb; spray voltage, 3.50 kV and −2.50 kV for ESI (+) and ESI (−), respectively; capillary temperature, 325 ℃; MS1 range, *m*/*z* 100–1000; MS1 resolving power, 70,000 FWHM; number of data-dependent scans per cycle, 10; MS/MS resolving power, 17,500 FWHM; normalized collision energy, 30 eV; dynamic exclusion time, automatic.

By comparing phenolic compounds' retention times and spectra to standards, phenolic compounds were found. The external calibration curve of each reference was used to quantify the characterized phenolic compounds. When the standard wasn't accessible, the compound quantification was defined as similar with the structurally closest phenolic compound.

### 2.5 The antioxidant assays of *R. roxburghii* DGs polyphenols

#### 2.5.1 DPPH radical scavenging activity

The DPPH of free and bound polyphenols from *R. roxburghii* DGs was evaluated using the method previously described with some modifications (Huang et al., 2022). In brief, 100 µL of extract solution (1:50 and 1:10 diluted with distilled water, respectively) was added to 1.9 mL DPPH solution (0.1 mmoL/L, 70% methanol solution), which was then reacted in the dark at 25 °C for 30 min and measured at 517 nm. The ability of DPPH was expressed as milligram of Trolox equivalent per milliliter of free and bound polyphenols from *R. roxburghii* DGs.

#### 2.5.2 ABTS radical scavenging activity

The ABTS activity of free and bound polyphenols from *R. roxburghii* DGs was measured using the method previously described with slight modifications (Huang et al., 2022). An equal volume of ABTS solution (7 mmol/L) and K_2_S_2_O_8_ (140 mmol/L) were mixed and incubated for 12–16 h. The mixture was diluted with ethanol to the absorbance of 0.90 ± 0.02 at 734 nm. Afterwards, 100 µL extract solution (1:20 diluted with distilled water) was added to 900 µL of the mixture and incubated for 30 min at 25 °C in dark. The absorbance was read at 734 nm. The ability of ABTS was expressed as milligram of Trolox equivalent per milliliter of free and bound polyphenols from *R. roxburghii* DGs.

#### 2.5.3 The ferric-reducing antioxidant power assay (FRAP)

The FRAP was measured according to a previous reference with some modifications (Cheng et al., 2020). The FRAP reagent comprised NaCl dissolved in acetic acid reagent (178 mmol/L), 10 mmol/L TPTZ dissolved in 40 mmol/L aqueous hydrochloric acid solution and 34 mmol/L FeCl_3_ at a ratio of 10:1:1. The 50 µL of extract solution (1:20 diluted with distilled water) and 2.45 mL of FRAP reagent were mixed and then the absorbance was measured at 593 nm. Data were reported as milligram of Trolox equivalent per milliliter of free and bound polyphenols from *R. roxburghii* DGs.

### 2.6 The *in vitro* digestion fermentation process

The *in vitro* assay was carried out according to the method of a previous studies (Cheng et al., 2020, Su et al., 2022). Gastric digestion: The pH of the polyphenol extract (1 mg/mL) was adjusted to 2.0 with 1 mol/L HCl. Subsequently, 280 µL of gastric solution (prepared with 72 mg/mL pepsin, 10,786 U) was added to the 10 mL polyphenol extract and incubated for 1 h in a shaking waterbath (180 rpm) at 37 °C.

Small intestinal digestion: At the end of the gastric stage, the pH of the digestive solution was immediately adjusted to 6.5 with 1 mol/L NaHCO_3_ and further to 7.4 with 1 mol/L NaOH. Next, an incubation period of 2 h at 37 °C with 180 rpm of shaking.

Colonic fermentation: The stool of two healthy volunteers (Female: 23 years old, BMI = 21.5; Male: 25 years old, BMI = 23.9.) who had not undergone antibiotic or prebiotic treatment within 3 months were respectively used for colonic fermentation. The fresh fecal sample was diluted with 0.01 mol/L sterilized PBS to make a 10% (w/v) fecal slurry. The 20 mL of fecal slurry and 100 mL of growth medium (peptone, 2 g/L; yeast extract, 2 g/L; NaCl, 0.1 g/L; K_2_HPO_4_, 0.04 g/L; KH_2_PO_4_, 0.04 g/L; NaHCO_3_, 2 g/L; MgSO_4_·7H_2_O, 0.01 g/L; CaCl_2_·6H_2_O, 0.01 g/L; tween 80, 2 mL/L; hemin, 50 mg/L; vitamin K,10 µL/L; l-cysteine, 0.5 g/L; bile salts, 0.5 g/L and resazurin, 1 mg/L) were mixed and the pH was maintained at 7.0 (Cheng et al., 2020). The low dosage (0.711 mL), medium dosage (1.420 mL) and high dosage (2.842 mL) of digested extract solution (0.56 mg/mL) were mixed with the fecal and growth medium mixture in the ratios of 1:18, 1:8 and 1:4 (v/v) in a 15 mL colonic system, respectively (Chan et al., 2023). The purity of free and bound polyphenols in *R. roxburghii* DGs ranged between 43.87 and 46.01% and 62.80 and 66.41%, respectively (Supplementary Table S4). Accordingly, the low, medium and high doses were calculated as 20, 70 and 140 mg/d of polyphenol content for humans during colonic fermentation (Chan et al., 2023, FDA, 2005, Lewis et al., 2018). The mixture was incubated anaerobically (90 % N_2_, 5% CO_2_ and 5% H_2_) at 37 °C for 24 h and 48 h. The mixture was centrifuged at 10, 000 rpm, 4 °C for 5 min to obtain pellets and supernatants, respectively storing at −20 °C for DNA extraction and SCFAs analysis.

### 2.7 Fecal microbial DNA extraction and qPCR

Fecal microbial DNA from colonic fermentation was extracted according to the manufacturer’s instructions. The concentration of extracted DNA was measured by absorbance at 260 nm, and the purity was evaluated by determining the A260/A280 and A260/A230 ratios using a NanoDrop ND-1000 spectrophotometer (Thermo Fisher Scientific, Waltham, MA, USA). The DNA integrity was measured by agarose gel electrophoresis (Supplementary Fig. S1.). Specific primers for fecal bacterial genera were synthesized by Sangon Biotech Co. Ltd. (Shanghai, China), including total bacteria, *Lactobacillus*, *Bifidobacterium*, *Ruminococcus*, *Akkermansia*, butyrate-producing bacteria, *Escherichia coli* and *Enterococcus*. The sequences of these primers were listed in Supplementary Table S2. The thermal cycling conditions were as follows: an initial denaturation step at 95 ℃ for 30 s, followed by 39 cycles of denaturation at 95 ℃ for 5 s, annealing at 53 ℃ for 30 s and extension at 72 ℃ for 30 s. The cycle threshold (Ct) was collected and the relative expression of each microbiota was recorded according to the 2^−ΔΔ Ct^ method, Δ Ct = Ct (target gene)-Ct (housekeeping gene); ΔΔ Ct = Δ Ct (experimental treatment)-Δ Ct (control treatment).

### 2.8 The SCFAs analysis

The SCFAs content was measured according to a previous reference with some modifications (Cheng et al., 2020). The colonic fermented supernatant (0.8 mL) was mixed with 160 µL H_2_SO_4_ (50%, v/v) for 10 min and the mixture was fully acidified for 1 h at 4 °C. Then 0.8 mL ethyl acetate was added and vortexed for 5 min. The mixture was incubated at 4 °C for 10 min and centrifuged at 13, 000 g for 5 min. Samples (1 µL) were analyzed at 1:10 spilt ratio by gas chromatography (GC 9720Plus, Fuli Instruments Co. Ltd., Zhejiang, China) on a DB-WAX chromatographic capillary column (30 m × 0.25 mm × 0.50 µm; Agilent, USA) under the following conditions: initial temperature of 105 °C for 3 min, heating to 170 °C at 10 °C/min and heating to 240 °C at 70 °C/min maintained for 2 min. The signal was detected at 250 °C with an FID detector.

### 2.9 Statistical analysis

Data are presented as the means ± standard deviations of triplicate experiments. Statistical significance was analyzed using one way analysis of variance followed by a post-hoc Duncan’s multiple range test (*p* < 0.05). Statistical analysis was performed by software SPSS 18 (IBM Co., USA). Redundancy analysis was performed and a Sankey diagram was imaged using the OmicStudio tools at https://www.omicstudio.cn/tool. Mantel test analysis was performed on Tutools platform at https://www.cloudtutu.com. Spearman’s correlation analysis was performed and images was generated with Origin 2023 (OriginLab, Northampton, MA, United States).

## 3 Results and discussion

### 3.1 Concentration of free polyphenols, bound polyphenols and total polyphenols

**Fig. 1:**
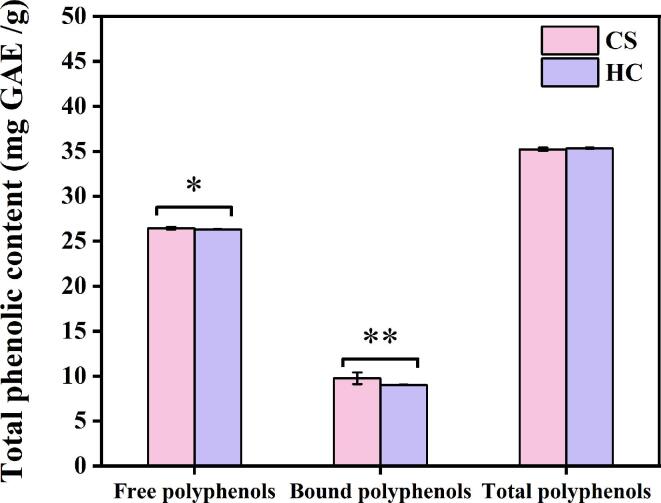
Content of phenolic compounds in *R. roxburghii* DGs. Including free polyphenols, bound polyphenols and total polyphenols, and the total phenolic content is the sum of free and bound fractions. Two kinds of *R. roxburghii* DGs named CS and HC were provided by Changshun Dnansoya *Rosa roxburghii* Farm Co. Ltd. (Guizhou, China) and Guizhou Hongcai Investment Group Co. Ltd. (Guizhou, China), respectively. Results were expressed as means ± SD, n = 3, *: *p* < 0.05, **: *p* < 0.01.

Fig. 1 showed the concentration of free polyphenols, bound polyphenols and total polyphenols of two kinds of *R. roxburghii* DGs. The free phenolic contents of CS and HC were 26.45 ± 14.98 mg GAE/g and 26.32 ± 13.86 mg GAE/g, respectively, which were significantly higher than that of bound polyphenols (CS:8.76 ± 0.65 mg GAE/g, HC:9.01 ± 0.13 mg GAE/g). In line with previous studies, most of the phenolic compounds in fruits or vegetables were dominated by the free forms, while bound polyphenols was combined with dietary fiber in plants via the ester-or ether-bond (Chan et al., 2023, Gomez-Mejia et al., 2019; Yang. et al., 2022). This study investigated the levels of fiber in *R. roxburghii* DGs (CS:49.78 g/100 g, HC:56.98 g/100 g) (Supplementary Table S1), as well as the bound polyphenols contributed to 24.88% and 25.50% of total polyphenols in CS and HC, respectively, indicating that there also existed numerous bound polyphenols in addition to the free polyphenols in *R. roxburghii* DGs. In addition, the total phenolic contents of CS and HC were 35.21 ± 19.01 mg GAE/g and 35.33 ± 18.09 mg GAE/g, respectively, which suggested that *R. roxburghii* DGs were indeed a valuable source of phenolic compounds compared to other foods. These values, for example, were greater than wine lees (23.17 mg GAE/g) (Jurčević et al., 2017), and comparable to phenolic-rich berry residues like grape (1.25 mg GAE/g) (Brezoiu et al., 2019) or blueberry (35.17 mg GAE/g) (Cladis et al., 2021). This result was consistent with previous studies wherein a total phenolic content of *R. roxburghii* pomace ranging from 8.57 to 45.40 mg GAE/g (Huang et al., 2022, Jiang et al., 2022, Li et al., 2022b, Xu et al., 2019). These results demonstrate that *R. roxburghii* DGs contain abundant phenolic compounds and provide the foundation to high value applications.

### 3.2 Identification and quantification of free and bound phenolic compounds

UPLC-ESI/QE-MS/MS was employed to identify and quantify the compositions of phenolic compounds (Supplementary Table S2). A total of 82 phenolic compounds, including 72 flavonoids, 8 phenolic acids and 2 tannins, were identified in free and bound polyphenols. Moreover, 32 individual phenolic compounds were quantitatively examined (Table 1). The results showed that abundant individual phenolic compounds of free polyphenols in CS and HC were catechin, taxifolin, quercitrin and myricetin, especially catechin (CS: 30466.33 ± 1365.32 mg/100 g, HC: 14529.82 ± 363.32 mg/100 g). In contrast, catechin, epicatechin, fisetin and procyanidin B2 were mainly found in bound polyphenols, especially the phenolic acid compounds such as gallic acid and salicylic acid in which were significantly higher than free polyphenols.

**Table 1:** Composition of phenolic compounds in *R. roxburghii* DGs.

No.	Phenolic compound	Rt(min)	Formula	Found at *m*/*z*	Expected at *m*/*z*	ppm	Free phenolic (mg//100 g)		Bound phenolic (mg//100 g)	
							CS	HC	CS	HC
1	Quercetin	10.135	C_15_H_10_O_7_	301.0347	302.0427	2.3	375.14 ± 18.97^a^	25.56 ± 21.45^b^	3.88 ± 0.31^b^	4.13 ± 1.05^b^
2	Quercitrin	5.695	C_21_H_20_O_11_	429.0830	448.1006	1.8	352.42 ± 107.5^a^	653.3 ± 651.61^a^	4.88 ± 0.39^b^	7.03 ± 5.85^b^
3	Taxifolin	7.632	C_15_H_12_O_7_	303.0510	304.0583	0.1	590.70 ± 499.71^a^	965.43 ± 95.28^a^	28.58 ± 4.29^b^	30.10 ± 18.17^b^
4	Fisetin	10.250	C_15_H_10_O_6_	287.0545	286.0480	2.7	82.76 ± 6.23^b^	169.49 ± 23.25^b^	1311.43 ± 123.41^a^	148.13 ± 52.95^b^
5	Catechin	6.420	C_15_H_14_O_6_	289.0715	290.0790	0.0	30466.33 ± 1365.32^a^	14529.82 ± 363.32^b^	2509.64 ± 36.85^c^	2533.82 ± 90.41^c^
6	Epicatechin	7.097	C_15_H_14_O_6_	291.0857	290.0790	2.0	19.21 ± 5.37^c^	20.58 ± 6.81^c^	2914.22 ± 88.89^a^	307.02 ± 193.23^b^
7	Daidzin	12.665	C_21_H_20_O_9_	397.2255	416.1107	2.4	22.05 ± 8.12^a^	18.25 ± 5.44^a^	16.10 ± 6.76^a^	19.15 ± 2.97^a^
8	Daidzein	7.070	C_15_H_10_O_4_	255.0648	254.0579	1.5	3.78 ± 0.04^c^	6.38 ± 1.64^b^	10.20 ± 1.1^a^	7.28 ± 1.2^b^
9	Glycitein	6.740	C_16_H_12_O_5_	285.0756	284.0685	0.0	4.41 ± 2.88^b^	8.55 ± 0.97^a^	0.17 ± 0.18^c^	0.41 ± 0.13^c^
10	Puerarin	6.612	C_21_H_20_O_9_	415.1044	416.1107	2.4	123.16 ± 14.64^b^	141.52 ± 5.86^a^	4.57 ± 0.34^d^	22.65 ± 0.46^c^
11	Myricetin	6.303	C_15_H_10_O_8_	319.0448	318.0376	0.1	6.74 ± 8.81^c^	10.52 ± 8.03^c^	390.26 ± 34.88^a^	58.15 ± 4.43^b^
12	Delphinidin 3-glucoside	6.880	C_21_H_21_O_12_	465.1014	465.1033	4.1	28.27 ± 6.89^b^	58.21 ± 4.19^a^	6.25 ± 4.05^c^	39.77 ± 26.47^a^
13	Kaempferol	8.262	C_15_H_10_O_6_	285.0396	286.0477	0.0	117.97 ± 49.93^a^	104.95 ± 8.28^a^	120.23 ± 6.28^a^	26.48 ± 16.71^b^
14	Astragalin	6.977	C_21_H_20_O_11_	429.0831	448.1006	2.1	92.82 ± 6.10^b^	380.2 ± 21.56^a^	0.19 ± 0.10^c^	0.51 ± 0.59^c^
15	Baicalin	7.957	C_21_H_18_O_11_	429.0814	446.0849	0.0	3.93 ± 0.18^b^	5.79 ± 0.49^b^	68.98 ± 18.83^a^	49.07 ± 35.19^a^
16	Cyanidin	8.445	C_15_H_11_O_6_	287.0558	287.0556	0.7	52.62 ± 2.94^a^	19.33 ± 0.83^b^	1.39 ± 0.14^c^	0.38 ± 0.03^c^
17	Apigenin	6.433	C_15_H_10_O_5_	271.0598	270.0528	1.0	2.76 ± 1.52^a^	3.80 ± 0.27^a^	3.23 ± 1.23^a^	0.95 ± 0.42^b^
18	Naringin	6.305	C_27_H_32_O_14_	563.1699	580.1792	3.3	259.98 ± 20.15^b^	437 ± 25.89^a^	3.10 ± 0.92^c^	2.91 ± 0.58^c^
19	Naringenin	10.073	C_15_H_12_O_5_	271.0609	272.0685	0.3	142.31 ± 2.90^b^	628.82 ± 12.95^a^	1.60 ± 0.21^c^	18.16 ± 12.70^c^
20	(2S)-Liquiritigenin	5.768	C_15_H_12_O_4_	257.0805	256.0736	1.5	9.65 ± 3.38^a^	12.38 ± 2.37^a^	1.52 ± 0.22^b^	0.66 ± 0.07^b^
21	Genistein	12.523	C_15_H_10_O_5_	269.0449	270.0528	1.2	7.14 ± 0.01^b^	15.35 ± 3.69^a^	5.64 ± 0.67^b^	1.78 ± 0.05^c^
22	Genistin	6.592	C_21_H_20_O_10_	431.0984	432.1056	0.2	17.71 ± 7.62^a^	13.01 ± 2.21^a^	0.77 ± 0.09^b^	0.8 ± 0.05^b^
23	Peonidin	13.820	C_16_H_13_O_6_	300.2622	301.2708	4.4	7.27 ± 5.3^a^	8.56 ± 4.08^a^	4.16 ± 2.66^a^	4.95 ± 2.26^a^
24	Luteolin	9.228	C_15_H_10_O_6_	287.0548	286.0477	0.6	3.79 ± 0.72^d^	13.01 ± 0.07^c^	16.04 ± 0.47^b^	20.23 ± 0.69^a^
25	Isorhamnetin	10.457	C_16_H_12_O_7_	315.0512	316.0583	0.6	1.87 ± 0.85^a^	2.38 ± 0.07^a^	0.03 ± 0.01^b^	0.01 ± 0^b^
26	Biochanin A	13.785	C_16_H_12_O_5_	283.1698	284.0685	1.1	8.19 ± 0.86^a^	9.12 ± 1.54^a^	9.95 ± 6.99^a^	2.69 ± 0.18^a^
27	Rutin	7.460	C_27_H_30_O_16_	611.1626	610.1534	1.6	95.41 ± 13.92^a^	48.95 ± 1.90^b^	1.01 ± 0.49c	11.51 ± 5.08c
28	Vanillin	4.987	C_8_H_8_O_3_	153.0549	152.0473	1.3	0.33 ± 0.01^b^	0.57 ± 0.03^a^	0.07 ± 0.00^c^	0.06 ± 0.00^c^
29	Salicylic acid	5.373	C_7_H_6_O_3_	137.0238	138.0317	2.7	16.29 ± 13.3^a^	16.13 ± 1.22^a^	4.24 ± 3.03^a^	4.17 ± 2.26^a^
30	Gallic acid	8.552	C_7_H_6_O_5_	169.0144	170.0215	1.0	2.4 ± 0.23^b^	38.85 ± 12.79^a^	3.47 ± 2.94^b^	12.16 ± 7.00^b^
31	Ellagic acid	13.583	C_14_H_6_O_8_	300.9984	302.0063	2.0	ND	ND	0.02 ± 0.01^b^	0.13 ± 0^a^
32	Procyanidin B2	5.495	C_30_H_26_O_12_	579.1468	578.1424	3.5	23.46 ± 35.07^a^	4.87 ± 0.82^a^	1.39 ± 1.61^a^	1.95 ± 1.88^a^

Two kins of *R. roxburghii* DGs named CS and HC were provided by Changshun Dnansoya *Rosa roxburghii* Farm Co. Ltd. (Guizhou, China) and Guizhou Hongcai Investment Group Co. Ltd. (Guizhou, China), respectively. Results were expressed as means ± SD, n = 3. Means with different letters were significantly different at *p* < 0.05.

Compared with the polyphenols in *R. roxburghii* fruit, which were mainly composed of macropolymers such as rutin, anthocyanin and glycoside (Liu et al., 2016), *R. roxburghii* DGs was generally composed of small phenolic compounds, like quercetin, catechin, naringin, gallic acid and chlorogenic acid. This was attributed to the fermentation of brewing microbiota (yeast strain), which promoted the metabolism of macromolecular phytochemicals to minor molecules during the brewing process of *R. roxburghii* (Shakour et al., 2020). Similarly, as previously proposed, quercetin 3-glucoside was degraded into quercetin during the brewing process (*Aspergillus awamori*) due to its greater hydrophilic properties than quercetin, which encouraged the accumulation of quercetin in *R. roxburghii* DGs (Lin et al., 2014). This result implies that the phenolic concentration in raw materials were significantly increased by the process of brewing *R. roxburghii* wine, due to yeast's ability to metabolize *R. roxburghii* fruit. Noteworthily, small phenolic compounds exert better antioxidant activities as they're more readily absorbed by the intestine and take part in metabolism than large phenolic compounds (Cheng et al., 2020).

Naturally, the *R. roxburghii* DGs still contained a variety of glycosidic macromolecular polyphenolic chemicals, which could improve intestinal health when linked with colonic microbiota. Therefore, for evaluating intestinal health and creating functional meals, it is crucial to investigate the potential functional qualities of phenolic compounds in *R. roxburghii* DGs.

### 3.3 The antioxidant activity of *R. roxburghii* DGs polyphenols

**Fig. 2:**
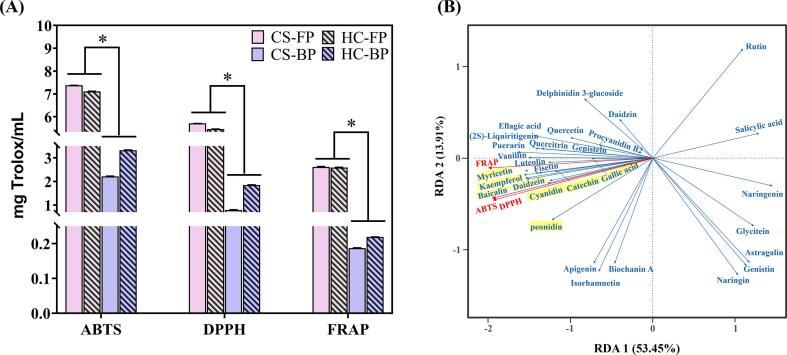
The antioxidant activities of *R. roxburghii* DGs. (A) The antioxidant activity of the various phenolic extracts of *R. roxburghii* DGs, including FRAP value, ABTS and DPPH free radical scavenging capacity. The free and bound polyphenols from CS (Changshun Dnansoya *Rosa roxburghii* Farm Co. Ltd) were named as CS-FP and CS-BP; the free and bound polyphenols from HC (Guizhou Hongcai Investment Group Co. Ltd.) were named as HC-FP and HC-BP, respectively. Results were expressed as means ± SD, n = 3, *: *p* < 0.05. (B) Redundancy analysis of phenolic contents and antioxidant activity in *R. roxburghii* DGs. The red vectors represent the antioxidant ability, the length of the line shows the correlation between the ranking axis and the magnitude, and the quadrant in which they are located reflects the positive and negative correlation between the antioxidant and the ranking axis. The blue vectors stand for the individual phenolic compounds of *R. roxburghii* DGs polyphenols, and their angle with the red vector denotes their relationship to the antioxidant. (For interpretation of the references to color in this figure legend, the reader is referred to the web version of this article.)

FRAP value, ABTS and DPPH free radical scavenging capacity were used to investigate the antioxidant activity of the various phenolic extracts of *R. roxburghii* DGs (Fig. 2A). The ABTS (7.11–7.38 mg Trolox/mL) and DPPH (5.46–5.71 mg Trolox/mL) values of free polyphenols were significantly (*p* < 0.05) higher than those of bound polyphenols (ABTS: 2.22–3.33 mg Trolox/mL, DPPH: 0.79–1.85 mg Trolox/mL), indicating that the radical scavenging activity of free polyphenols was superior to that of bound polyphenols. These results were comparable to the *R. roxburghii* dry fruit (DPPH:5.05 mg AAE/g) (Yan et al., 2022) and greater than phenolic extracts from popular fruit remnants like citrus (1.40 mg Trolox/g) (Gomez-Mejia et al., 2019) and lemons (1.10 mg TE/g) (Papoutsis et al., 2016). It was unavoidable that it was lower than the *R. roxburghii* fresh fruit phenolic extract (ABTS: 8.39–9.32 mmol Trolox/L, DPPH: 4.45–6.00 mmol Trolox/L) (Jiang et al., 2022), though, as some polyphenol ingredients will be degraded in the wine-brewing process (Arfaoui, 2021). Moreover, FRAP analysis (free polyphenols: 2.60–2.61 mg Trolox/mL, bound polyphenols: 0.19–0.22 mg Trolox/mL) served to confirm the results. Based on those, it was reasonable to believe that the *R. roxburghii* DGs polyphenols, both free and bound, have potential antioxidant effects.

Redundancy analysis was thus applied in this study to examine which individual phenolic compounds are the keystone that contribute greatly to antioxidant activity in both the free and bound polyphenols of *R. roxburghii DG*s. As shown in Fig. 2B, the first two principal components could load the most information from the original data, as they accounted for 67.36% of the total variability in the *R. roxburghii* DGs polyphenols. The antioxidant activity was strongly related to the *R. roxburghii* DGs polyphenols (r^2^ = 0.98–0.99, *p* < 0.01) and free polyphenols were better than bound polyphenols. It could be that bound polyphenols linked to fiber in plants were difficult to release, whereas free polyphenols were soluble in water or polar solvents and were easily to liberate electrons or hydrogen atoms, contributing to the high free radical scavenging ability. Hence, free polyphenols were the primary antioxidant in fruits and vegetables in line with previous research (Mithul Aravind et al., 2021, Yang et al., 2022).

Moreover, the mean value (RDA1: 0.74, RDA2: 0.27, RDA3: 0.10) of peonidin, daidzein, kaempferol, baicalin and myricitrin was greater than that of other individual phenolic compounds, confirming that they were key individual phenolic compounds in the antioxidant function of *R. roxburghii* DGs (Fig. 2B). Peonidin, an anthocyanidin family member, was a well-known antioxidant found in berries that prevented oxidative reactions by competing with free radicals (Rajan et al., 2018). Daidzein, an isoflavone with two phenolic hydroxyl groups that are highly antioxidants, shielded IPEC-J2 cells from H_2_O_2_-induced oxidative stress and may positively affect intestinal function (Li et al., 2022c). Kaempferol and myricitrin, flavonol components of plants, have improved the integrity of intestinal epithelial cells and intestinal barrier dysfunction *in vivo*. (Jin et al., 2021). Baicalin was a natural flavonoid glycoside that attenuated intestinal oxidative stress by ameliorating the peroxidation of free radicals or enhancing the activity of antioxidant enzymes (Wang et al., 2022). Notably, catechin and gallic acid, the most abundant flavonoids and phenolic acids in *R. roxburghii* DGs polyphenols, also made a major contribution to the phenolic antioxidant activity (Fig. 2B). Furthermore, cyanidin, in its capacity as a strong antioxidant, had an RDA1 value of 0.67 in this study (Fig. 2B), indicating that it played a key role in the antioxidant ability of *R. roxburghii* DGs polyphenols. Thus, *R. roxburghii* DGs polyphenols have the potential to regulate intestinal oxidative stress levels, providing an excellent foundation for the regulation of intestinal microbiota by *R. roxburghii* DGs.

### 3.4 Modulation of fecal microbiota by *R. roxburghii* DGs polyphenols

**Fig. 3:**
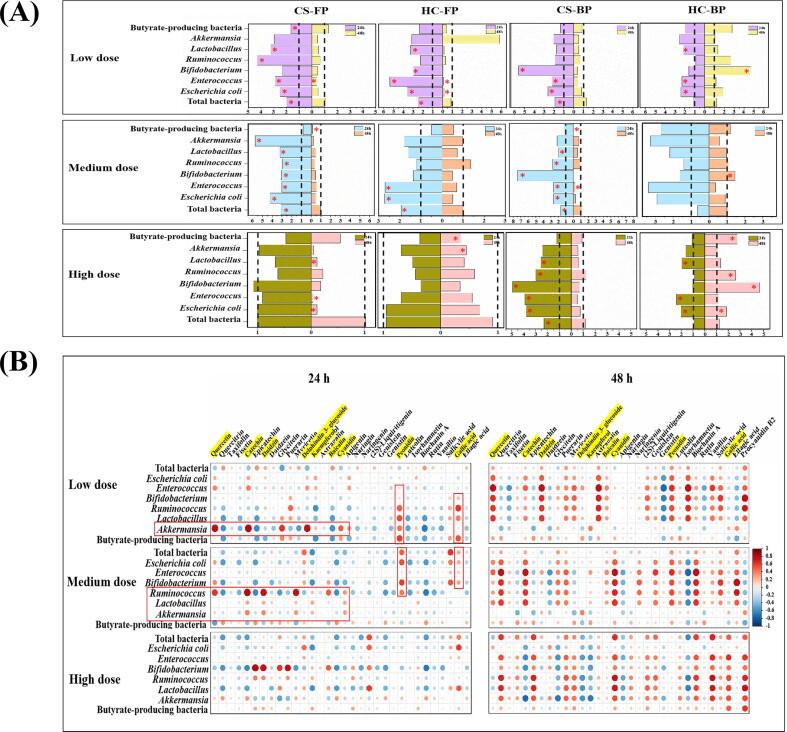
The modulatory effects of *R. roxburghii* DGs polyphenols on fecal microbiota. (A) The relative abundance of fecal microbiota in *R. roxburghii* DGs polyphenols treatment after the colonic fermentation. Fermentation for 0 h were set as the corresponding control to evaluate the relative effect of low (20 mg/d), medium (70 mg/d) and high (140 mg/d) doses of CS and HC *R. roxburghii* DGs polyphenols treatments on fecal microbiota, respectively. Means were higher or lower than 1.00, revealing that the treatment increased or decreased the abundance of specific fecal microbiota, respectively. (B) Correlation analysis of phenolic contents and the relative abundance of fecal microbiota in *R. roxburghii* DGs. The free and bound polyphenols from CS (Changshun Dnansoya *Rosa roxburghii* Farm Co. Ltd) were named as CS-FP and CS-BP; the free and bound polyphenols from HC (Guizhou Hongcai Investment Group Co. Ltd.) were named as HC-FP and HC-BP, respectively. Results were expressed as means ± SD, n = 3, *: *p* < 0.05.

The qPCR was used to investigate the effect of polyphenols from *R. roxburghii* DGs on the abundance of fecal microbiota *in vitro* fermentation (Fig. 3A). After the *in vitro* fermentation for 24 h, the abundance of *Bifidobacterium*, *Ruminococcus*, *Lactobacillus* and *Akkermansia* was significantly increased (*p* < 0.05) by free polyphenols of *R. roxburghii* DGs (Purity: 43.87–46.01%; 20–70 mg/d) compared to the control, especially the treatment of free polyphenols from CS sample (20 mg/d), which significantly increased (*p* < 0.05) to 3.05 ± 0.54-, 2.96 ± 0.04-, 3.21 ± 0.21- and 5.74 ± 0.67-fold, respectively. Moreover, the bound polyphenols of CS (Purity: 66.41%; 70–140 mg/d) significantly (*p* < 0.05) promoted *Bifidobacterium*, *Ruminococcus* and *Lactobacillus*, as well as *Akkermansia* with butyrate-producing bacteria, although insignificantly (*p* > 0.05). While the abundance of *Escherichia coli* was moderately increased *in vitro* fermentation for 24 h, in addition to probiotics. This could be a result of the gut microbiome being dynamic, with beneficial microorganisms, conditionally pathogenic microorganisms, symbiotic microorganisms and harmful microorganisms all changing dynamically to reveal a dynamic equilibrium (Das & Nair, 2019) The bound polyphenols of HC (Purity: 62.80%) promoted *Bifidobacterium*, butyrate-producing bacteria and *Ruminococcus* after colonic fermentation for 48 h, significantly (*p* < 0.05). The growth of *Escherichia coli* and *Enterococcus* was inhibited in free polyphenols and bound polyphenols from CS sample in 48 h-fermentation. It was notable that the abundance of probiotics was also were reduced during *in vitro* fermentation for 48 h. This appears to be because, during the simulated fermentation process, the bacteria consumed specific nutrients for the growth of themselves, and those nutrients became depleted as the fermentation period increased (Silva et al., 2022). Nonetheless, beneficial bacteria (*Akkermansia*, *Lactobacillus*, *Ruminococcus* and *Bifidobacterium*) remained prevalent in the intestinal environment based on their general effect on fecal bacteria.

Sufficient evidences showed that dietary polyphenols consumption influences the composition of gut microbiota. *Akkermansia* and *Ruminococcus* were reported as mucosa-associated bacteria, which can stabilize the structure of the mucus layer and maintain intestinal barrier function (Lee et al., 2018). Consistent with the present study, *Ruminococcus* and *Akkermansia* levels were increased in high-fat diet-fed mice after 8 weeks of supplying *R. roxburghii* fruit phenolic extracts (400 mg/kg body weight) (Wang et al., 2022, Wang et al., 2022). *Bifidobacterium* and *Lactobacillus* were also important for gut health because they motivate immune function, modulate metabolic reactions and inhibit pathogenic bacteria. A similar pattern of results was obtained in *Lycium ruthenicum*, where phenolic compounds increased the abundance of *Lactobacillus* and *Bifidobacterium* (Luo et al., 2019)*.* In addition, lactic acid produced by *Bifidobacterium* and *Lactobacillus* in the colonic fermentation was used to produce butyrate by butyrate-producing bacteria (*Roseburia*, *Blautia* and *Coprococcus*), which play a role in anti-inflammatory and modulation in the gut (Fan & Pedersen, 2021). The conclusion was consistent with research showing that bound polyphenols from *R. roxburghii* pomace improved increased the abundance of butyrate-producing bacteria and SCFAs production (Su et al., 2022). According to numerous studies, *Escherichia coli* and *Enterococcu*s were potentially pathogenic bacteria that damaged intestinal health (Cheng et al., 2020, Fan and Pedersen, 2021). In the present study, the free and bound polyphenols of CS strongly inhibited the development of these two bacteria (Fig. 3A). Overall, it might conclude that *R. roxburghii* DGs polyphenols are favorable for the growth of beneficial bacteria as well as the prevention of bacteria with conditional pathogenicity, with free polyphenols from CS sample *in vitro* colonic fermentation for 24 h exhibiting the best results.

Spearman’s correlation analysis was performed to further investigate which individual phenolic compounds played the main role in effect of the fecal microbiota in *R. roxburghii* DGs (Fig. 3B). In 24 h-fermented samples (70 mg/d), quercetin, catechin, daidzin and peonidin significantly impacted *Ruminococcus* (r^2^ = 0.64–0.82, *p* < 0.05) production. Delphinidin 3-glucoside and above compounds were similarly strongly associated with the enrichment of *Akkermansia* (r^2^ = 0.78–0.82, *p* < 0.05) at *R. roxburghii* DGs polyphenols (20 mg/d), demonstrating that these compounds were crucial to the growth of *Akkermansia* and *Ruminococcus*. However, *R. roxburghii* DGs polyphenols did appear to suppress microbial growth at 140 mg/d, which may be related to the phenomenon of polyphenol saturation (Renouf et al., 2013). Quercetin, a natural flavonol, has been shown to optimize intestinal homeostasis and keep the gut healthy by activating beneficial bacteria and preventing potentially pathogenic bacteria (Shi et al., 2020). Due to their complex structures, the flavonoids catechin, daidzin and delphinidin-3-glucoside have contributed to modifying the composition and structure of the gut microbiota (Mithul Aravind et al., 2021). As evidenced in recent study, consumption of daidzin could promote the establishment of probiotics such as *Bifidobacterium*, *Lactobacillus* and *Akkermansia* (Li et al., 2022c). Moreover, the improvement in *Akkermansia, Lactobacillus, Ruminococcus* and *Bifidobacterium* was strongly correlated with peonidin and gallic acid, which has been demonstrated in previous studies (Yang et al., 2020). However, it was notable that kaempferol, cyanidin and baicalin (r^2^ = 0.19–0.51), which improved the antioxidant abilities of *R. roxburghii* DGs polyphenols (Fig. 2B), were also favorable for the growth of beneficial bacteria (*Ruminococcus*, *Lactobacillus*, *Akkermansia* and butyrate-producing bacteria) (Fig. 3B). This suggested that these phenolic compounds might also be implicated in the health effects underlying the modulation of fecal microbiota by *R. roxburghii* DGs polyphenols. Moreover, the concentration of SCFAs was regarded as evidence to further evaluate the effect of *R. roxburghii* DGs polyphenols on fecal microbiota.

### 3.5 Effect of *R. roxburghii* DGs polyphenols on SCFAs production

**Fig. 4:**
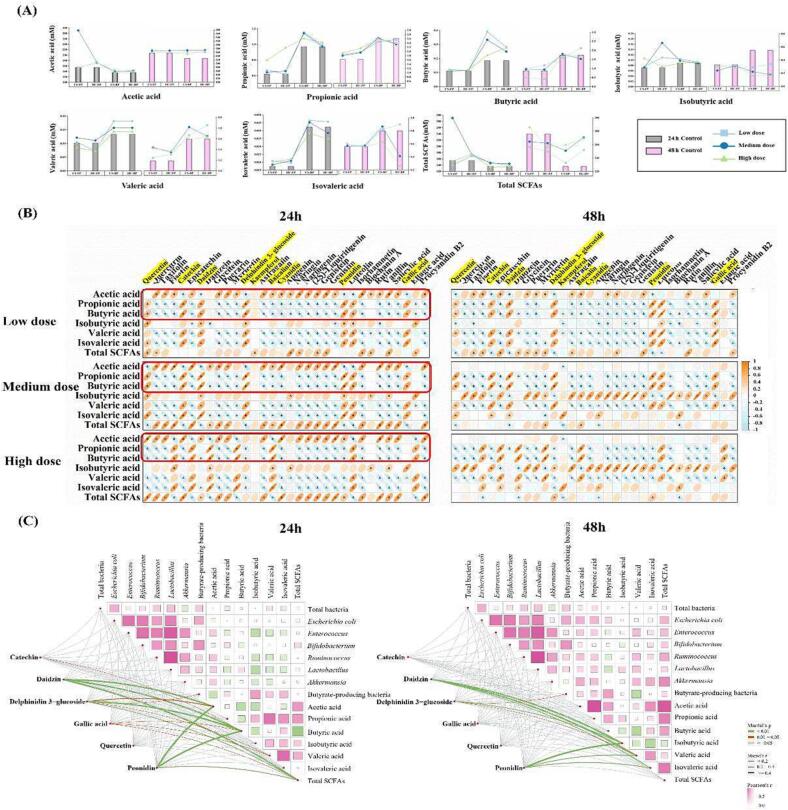
The effect of *R. roxburghii* DGs polyphenols on SCFAs production. (A) Effects of *R. roxburghii* DGs polyphenol treatments on the concentration of SCFAs in the simulated colon fermentation system. This includes acetic acid, propionic acid, butyric acid, isobutyric acid, valeric acid, isovaleric acid, and total SCFAs. The bar chart represents the control group, and the broken line chart represents the sample group. (B) Correlation analysis of phenolic contents and acetic acid, propionic acid, butyric acid, isobutyric acid, valeric acid, isovaleric acid, and total SCFAs in *R. roxburghii* DGs. Results were expressed as means ± SD, n = 3, *: *p* < 0.05. (C) Relationships between contents of phenolic compound of *R. roxburghii* DGs, relative abundance of fecal microbiota and concentration of SCFAs composition in the simulated colon fermentation system. Comparisons of relative abundance of fecal microbiota and concentration of SCFAs composition in the simulated colon fermentation system are shown, with a color gradient denoting. Spearman’s correlation coefficients. The contents of phenolic compound of *R. roxburghii* DGs were related to each environmental factors by mantel test.

The results demonstrated that the free and bound polyphenols of *R. roxburghii* DGs promoted the production of total SCFAs (Fig. 4A), which was useful for the modulation of gut health, especially the promotion of acetic acid, propionic acid and butyric acid for 24 h-fermentation under the simulated colonic fermentation system (Fig. 4A). Acetic acid, propionic acid and butyric acid, accounting for about 90% of the total SCFAs, were described as key regulators as they could contribute to gut health (Koh et al., 2016). According to the results, acetic acid made up the main share of the total SCFAs, and most was absorbed and used as food in the peripheral circulation (Koh et al., 2016). Compared with the control group, treatment with free polyphenols for 24 h-fermentation and bound polyphenols for 48 h-fermentation significantly (*p* < 0.05) promoted the increase of acetic acid, particularly with free polyphenols (70 mg/d) from CS sample being 290.87 mM (*p* < 0.01) (Fig. 4A). Propionic acid was useful for gut health by ameliorating metabolic disorders, reducing food-intake and inhibiting anti-inflammation (Koh et al., 2016). In this work, *R. roxburghii* DGs polyphenols generally promoted the production of propionic acid, especially the free polyphenols and bound polyphenols from CS sample, which showed significant effects (*p* < 0.05) at 24 h-fermentation (Fig. 4A). Butyric acid, which regulates the intestinal microbiota, protects the integrity of the intestinal mucosa and maintains the homeostasis of the intestinal environment (Macfarlane & Macfarlane, 2012). This result also showed promising findings, with butyric acid concentrations of 0.40 mM and 1.18 mM for free polyphenols from the CS sample (24 h-fermentation) and bound polyphenols from the HC sample (48 h-fermentation), respectively. Furthermore, the levels of branched-chain fatty acids such as isobutyric acid and isovaleric acid were rose (Fig. 4A). Despite their relatively modest levels, branched-chain fatty acids have been demonstrated to play a significant role in intestinal homeostasis (Koh et al., 2016). Hence, it was clear that *R. roxburghii* DGs polyphenols enhanced the production of SCFAs in the colonic fermentation system, providing evidence to effectively modulate intestinal microbiota.

Spearman's correlation analysis was used to further investigate the key individual phenolic compound on intestinal function. As shown in Fig. 4B, acetic acid showed positive correlations (*p* < 0.05) with quercetin (r^2^ = 0.89) and catechin (r^2^ = 0.85), while propionic acid and butyric acid positively correlated (*p* < 0.05) with quercetin (r^2^ = 0.64–0.69), catechin (r^2^ = 0.78–0.82), daidzin (r^2^ = 0.86–0.87), delphinidin 3-glucoside (r^2^ = 0.80–0.87), peonidin (r^2^ = 0.86–0.88) and gallic acid (r^2^ = 0.61–0.67). Importantly, daidzin, delphinidin 3-glucoside and peonidin were found to be positively correlated with (*p* < 0.01) acetic acid (r^2^ = 0.57, 0.21 and 0.43), butyric acid (r^2^ = 0.75, 0.49 and 0.66), and total SCFAs (r^2^ = 0.46, 0.38 and 0.30) for 24 h-fermentation (Fig. 4C). Similar to what was found in the previous study, it was shown that *Bifidobacterium*, *Ruminococcus*, *Lactobacillus* and *Akkermansia* all benefited from the 20–70 mg/d *R. roxburghii* DGs polyphenols for 24 h-fermentation (Fig. 3A). Similarly, quercetin, catechin and gallic acid were also found to have similar positive effect (Fig. 4C). Hence, the health effects underlying the modulation of *R. roxburghii* DGs polyphenols on the fecal microbiota were made clear. The acetic acid and propionic acid were produced by these beneficial bacteria metabolizing most bound polyphenols according to previous research (Macfarlane & Macfarlane, 2012). Moreover, lactic acid generated by *Lactobacillus* and *Bifidobacterium* could produce propionic acid or butyric acid with strict anaerobic bacteria by cross-feeding mechanisms, which increased the SCFAs production (Fan & Pedersen, 2021; Koh et al., 2016). Furthermore, butyric acid was generated by butyrate-producing bacteria using the bound polyphenols of *R. roxburghii* DGs by butyric kinase or butyryl CoA (Macfarlane and Macfarlane, 2012, Su et al., 2022). However, the promoting effect of *R. roxburghii* DGs polyphenols at 24 h-fermentation on the microbiota abundance and SCFAs concentration was better than 48 h. This appeared to be caused by a lack of energy, as these beneficial microbes produced energy by metabolizing the food substrate to support growth. If their inhibition occurred, the level of SCFAs in the colon would decrease (Koh et al., 2016). Therefore, it was sufficient to point out the main roles of quercetin, catechin, daidzin, delphinidin 3-glucoside, peonidin and gallic acid from *R. roxburghii* DGs in improving the growth of beneficial microorganisms.

**Fig. 5:**
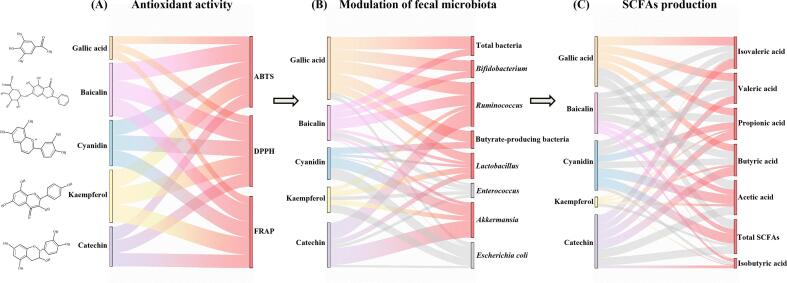
The health effects on gut health are supplied by the five important phenolic compounds from *R. roxburghii* DGs polyphenols, including quercetin, catechin, kaempferol, cyanidin and baicalin. (A) The decrease of intestinal oxidative stress. (B) The modulation of the fecal microbiota's community structure. (C) The increase of SCFAs concentration. The red color denotes the promoting effect, the gray color denotes the inhibiting impact, and the line thickness indicates the intensity of the effect. (For interpretation of the references to color in this figure legend, the reader is referred to the web version of this article.)

Additionally, acetic acid, propionic acid and butyric acid all showed positive associations with kaempferol, cyanidin and baicalin (Fig. 4B), which improved the antioxidant abilities of *R. roxburghii* DGs polyphenols (Fig. 2B). For example, baicalin (r^2^ = 0.48) and cyanidin (r^2^ = 0.71) showed positive correlations (*p* < 0.05) with acetic acid; kaempferol was positively correlated with the three major acids, although insignificantly (Fig. 4B). This confirmed that these phenolic compounds can modulate the fecal microbiota. As discussed, it can be concluded that quercetin, catechin, kaempferol, cyanidin and baicalin were the five important polyphenols that played a part in antioxidant activity and microbiota regulation of *R. roxburghii* DGs polyphenols (Fig. 5). Overall, the study showed that *R. roxburghii* DGs polyphenols had potential gut-improving functions by reducing intestinal oxidative stress levels, altering the fecal microbiota's community structure and raising the level of SCFAs content, revealing that the keystone individual phenolic compounds were quercetin, catechin, kaempferol, cyanidin and baicalin.

## 4 Conclusions

*R. roxburghii* DGs showed potential usage for the remained polyphenols, which enhanced antioxidant activity and improved gut health. Free polyphenols revealed higher antioxidant activities than bound one in *R. roxburghii* DGs. Moreover, both the free and bound polyphenols from *R. roxburghii* DGs improved the fecal microbiota community structure and accumulated SCFAs. Importantly, quercetin, catechin, kaempferol, cyanidin and baicalin were key compounds in demonstrating functional properties, revealing the health effects by which *R. roxburghii* DGs polyphenols moderated fecal microbiota. Overall, this study offered the theoretical basis for the high-value usage of *R. roxburghii* DGs and developing *R. roxburghii* DGs into functional food additives. This idea will provide effective reuse strategies and commercial benefits for the wine industry.

## CRediT authorship contribution statement

**Die Zhou:** Data curation, Writing. **Jiang Zhong:** Editing. **Yongguang Huang:** Editing, Methodology, Investigation. **Yuxin Cheng:** Funding acquisition, Resources, Methodology.

## Declaration of Competing Interest

The authors declare that they have no known competing financial interests or personal relationships that could have appeared to influence the work reported in this paper.

## Data Availability

Data will be made available on request.
