# Supplementary material for: Effect of free and bound polyphenols from Rosa roxburghii Tratt distiller's grains on moderating fecal microbiota
Source: Food Chem X. 2023 Jun 12;19:100747. doi: 10.1016/j.fochx.2023.100747 (PMC10534110; doi:10.1016/j.fochx.2023.100747)
Supplement: Supplementary data 1 [file mmc1.docx]

**Supplementary Table S1. Contents of basic components in *R. roxburghii DGs***

| Elementary component (g/100g) | CS | HC |
| --- | --- | --- |
| Moisture | 6.63 ± 0.11** | 11.23 ± 0.20 |
| Ash | 3.48 ± 0.17* | 2.77 ± 0.26 |
| Starch | 3.27 ± 0.01** | 3.20 ± 0.01 |
| Protein | 11.23 ± 0.20** | 7.58 ± 0.51 |
| Fat | 0.49 ± 0.10* | 0.70 ± 0.13 |
| Reducing sugar | 5.77 ± 0.09** | 6.99 ± 0.13 |
| Vitamin C | 0.02 ± 0.01 | 0.02 ± 0.01 |
| Titratable acid | 2.48 ± 0.09** | 2.66 ± 0.01 |
| Dietary fiber | 49.78 ± 0.16** | 56.98± 0.15 |

Two kins of *R. roxburghii* DGs named CS and HC were provided by Changshun Dnansoya *Rosa roxburghii* Farm Co. Ltd. (Guizhou, China) and Guizhou Hongcai Investment Group Co. Ltd. (Guizhou, China), respectively. Results were expressed as means ± SD, n = 3, *: *p* < 0.05, **: *p* < 0.01.

**Supplementary Table S2. Primers of specific fecal microbiota**

| Name | Primers | References |
| --- | --- | --- |
| Total bacteria | F: GCAGGCCTAACACATGCAAGTC  R: CTGCTGCCTCCCGTAGGAGT | (Xu et al., 2013) |
| *Escherichia coli* | F: GTTAATACCTTTGCTCATTA  R: ACCAGGGTATCTTAATCCTGTT | (Aloisio et al., 2012) |
| *Enterococcus* | F: CCCTTATTGTTAGTTGCCATCAT  R: ACTCGTTGTACTTCCCATTGT | (Rinttila et al., 2004) |
| *Bifidobacterium* | F: GGGTGGTAATGCCGGATG  R: TAAGCGATGGACTTTCACACC | (Louis & Flint, 2009) |
| *Ruminococcus* | F: TTAACACAATAAGTWATCCACCTGG  R: ACCTTCCTCCGTTTTGTCAAC | (Kennedy et al., 2014) |
| *Lactobacillus* | F: AGCAGTAGGGAATCTTCCA  R: CACCGCTACACATGGAG | (Gorzelak et al., 2015) |
| *Akkermansia* | F: CAGCACGTGAAGGTGGGGAC  R: CCTTGCGGTTGGCTTCAGAT | (Xu et al., 2013) |
| Butyrate-producing bacteria | F: GCIGAICATTTCACITGGAAYWSITGGCAYATG  R: CCTGCCTTTGCAATRTCIACRAANGC | (Louis et al., 2009) |
| Housekeeping gene | F: ACTCCTACGGGAGGCAGCAG  R: GGACTACHVGGGTWTCTAAT | (Xu. et al., 2016) |

F and R were forward primer and reverse primer, respectively.

**Supplementary Table S3. Composition of phenolic compounds in *R. roxburghii DGs***

| **No.** | **Phenolic compound** | **Rt(min)** | **Formula** | **Selected ion** | **CAS** | **Found at m/z** | **Expected at m/z** | **ppm** |
| --- | --- | --- | --- | --- | --- | --- | --- | --- |
|  | **Flavonoids** |  |  |  |  |  |  |  |
|  | Quercetin and its derivatives |  |  |  |  |  |  |  |
| 1 | Quercetin | 10.135 | C_15_H_10_O_7_ | [M-H]- | 117-39-5 | 301.0347 | 302.0427 | 2.3 |
| 2 | Quercitrin | 5.695 | C_21_H_20_O_11_ | [M-H2O-H]- | 522-12-3 | 429.0830 | 448.1006 | 1.8 |
| 3 | Isoquercitrin | 7.170 | C_21_H_20_O_12_ | [M+H]+ | 482-35-9 | 465.1017 | 464.0955 | 2.3 |
| 4 | Taxifolin | 7.632 | C_15_H_12_O_7_ | [M-H]- | 480-18-2 | 303.0510 | 304.0583 | 0.1 |
| 5 | Fisetin | 10.250 | C_15_H_10_O_6_ | [M+H]+ | 345909-34-4 | 287.0545 | 286.0480 | 2.7 |
| 6 | Pinoquercetin | 4.817 | C_16_H_12_O_7_ | [M+H]+ | 491-49-6 | 317.0648 | 316.0583 | 2.4 |
| 7 | Peracetate Quercetin | 6.122 | C_25_H_20_O_12_ | [M+H]+ | 1064-06-8 | 513.1007 | 512.0955 | 4.0 |
| 8 | 3,7-Dimethylquercetin | 6.055 | C_17_H_14_O_7_ | [M+H]+ | 2068-02-2 | 331.0808 | 330.0740 | 1.4 |
| 9 | Quercetin 4',7-diglucoside | 4.803 | C_27_H_30_O_17_ | [M-H]- | 42900-82-3 | 625.1394 | 626.1483 | 2.6 |
| 10 | Quercetin 3-arabinoside | 5.475 | C_20_H_18_O_11_ | [M-H2O-H]- | 22255-13-6 | 415.0664 | 434.0849 | 0.3 |
| 11 | Quercetin 3-(6''-acetylglucoside) | 4.812 | C_23_H_22_O_13_ | [M-H]- | 54542-51-7 | 505.0978 | 506.1060 | 1.9 |
|  | Catechin and its derivatives |  |  |  |  |  |  |  |
| 12 | Catechin | 6.420 | C_15_H_14_O_6_ | [M-H]- | 154-23-4 | 289.0715 | 290.0790 | 0.0 |
| 13 | Catechin pentaacetate | 6.873 | C_25_H_24_O_11_ | [M+H]+ |  | 501.1387 | 500.1319 | 0.8 |
| 14 | (-)-Catechin gallate | 8.253 | C_22_H_18_O_10_ | [M+H]+ | 130405-40-2 | 443.0974 | 442.0900 | 0.0 |
| 15 | Epicatechin | 7.097 | C_15_H_14_O_6_ | [M+H]+ | 490-46-0 | 291.0857 | 290.0790 | 2.0 |
| 16 | Epicatechin pentaacetate | 7.543 | C_25_H_24_O_11_ | [M+H]+ |  | 501.1408 | 500.1319 | 3.3 |
| 17 | Epigallocatechin gallate | 5.458 | C_22_H_18_O_11_ | [M-H]- | 989-51-5 | 457.0776 | 458.0849 | 0.1 |
|  | Soy isoflavone |  |  |  |  |  |  |  |
| 18 | Daidzin | 12.665 | C_21_H_20_O_9_ | [M-H2O-H]- | 552-66-9 | 397.2255 | 416.1107 | 2.4 |
| 19 | Daidzein | 7.070 | C_15_H_10_O_4_ | [M+H]+ | 486-66-8 | 255.0648 | 254.0579 | 1.5 |
| 20 | Ononin | 8.878 | C_22_H_22_O_9_ | [M+H]+ | 486-62-4 | 431.1311 | 430.1264 | 0.5 |
| 21 | Glycitin | 6.803 | C_22_H_22_O_10_ | [M+H]+ | 40246-10-4 | 447.1266 | 446.1213 | 4.4 |
| 22 | Glycitein | 6.740 | C_16_H_12_O_5_ | [M+H]+ | 40957-83-3 | 285.0756 | 284.0685 | 0.0 |
| 23 | Equol | 5.873 | C_15_H_14_O_3_ | [M+H-H2O]+ | 531-95-3 | 225.0870 | 242.0943 | 0.6 |
| 24 | Puerarin | 6.612 | C_21_H_20_O_9_ | [M-H]- | 3681-99-0 | 415.1044 | 416.1107 | 2.4 |
|  | Myricetin and its derivatives |  |  |  |  |  |  |  |
| 25 | Myricetin | 6.303 | C_15_H_10_O_8_ | [M+H]+ | 529-44-2 | 319.0448 | 318.0376 | 0.1 |
| 26 | Myricitrin | 6.577 | C_21_H_20_O_12_ | [M-H]- | 17912-87-7 | 463.0870 | 464.0955 | 2.6 |
| 27 | Myricetin 3-galactoside | 12.115 | C_21_H_20_O_13_ | [M]+ | 15648-86-9 | 480.3738 | 480.3757 | 4.0 |
| 28 | myricetin 3-O-beta-D-galactopyranoside | 5.430 | C_21_H_20_O_13_ | [M-H]- |  | 479.0810 | 480.0904 | 4.4 |
|  | Delphin and its derivatives |  |  |  |  |  |  |  |
| 29 | Leucodelphinidin | 7.090 | C_15_H_14_O_8_ | [M+H]+ |  | 323.0761 | 322.0689 | 0.1 |
| 30 | Delphinidin 3-glucoside | 6.880 | C_21_H_21_O_12_ | [M]- | 6906-38-3 | 465.1014 | 465.1033 | 4.1 |
| 31 | Delphinidin 3-(6-p-coumaroyl)glucoside | 8.368 | C_30_H_27_O_14_ | [M]- |  | 611.1381 | 611.1401 | 3.3 |
| 32 | delphinidin-3-O-arabinoside | 6.193 | C_20_H_19_O_11_ | [M]+ |  | 435.0897 | 435.0927 | 4.8 |
| 33 | Delphinidin 3-O-beta-D-sambubioside | 5.768 | C_26_H_29_O_16_ | [M+H-H2O]+ | 53158-73-9 | 580.1378 | 597.1456 | 0.6 |
|  | Kaempferol and its derivatives |  |  |  |  |  |  |  |
| 34 | Kaempferol | 8.262 | C_15_H_10_O_6_ | [M-H]- | 520-18-3 | 285.0396 | 286.0477 | 0.0 |
| 35 | Kaempferitrin | 5.697 | C_27_H_30_O_14_ | [M-H2O-H]- | 482-38-2 | 559.1445 | 578.1636 | 1.3 |
| 36 | Astragalin | 6.977 | C_21_H_20_O_11_ | [M-H2O-H]- | 480-10-4 | 429.0831 | 448.1006 | 2.1 |
| 37 | Baicalin | 7.957 | C_21_H_18_O_11_ | [M+H-H2O]+ | 21967-41-9 | 429.0814 | 446.0849 | 0.0 |
| 38 | 6''-O-p-Coumaroyltrifolin | 5.853 | C_30_H_26_O_13_ | [M-H2O-H]- | 68170-52-5 | 575.1187 | 594.1373 | 0.4 |
| 39 | Kaempferol 3-O-beta-D-xyloside | 7.473 | C_20_H_18_O_10_ | [M+H]+ |  | 419.0968 | 418.0900 | 1.0 |
| 40 | Kaempferol 3,7-diglucoside | 15.208 | C_27_H_30_O_16_ | [M-H]- |  | 609.5075 | 610.5175 | 4.5 |
|  | Cyanidin and its derivatives |  |  |  |  |  |  |  |
| 41 | Cyanidin | 8.445 | C_15_H_11_O_6_ | [M]- | 528-58-5 | 287.0558 | 287.0556 | 0.7 |
| 42 | Leucocyanidin | 4.272 | C_15_H_14_O_7_ | [M]+ | 93527-39-0 | 306.0988 | 306.0740 | 4.0 |
| 43 | Cyanidin 3-glucoside | 7.627 | C_21_H_21_O_11_ | [M]+ | 7084-24-4 | 449.1081 | 449.1084 | 0.7 |
| 44 | Cyanidin 3-rutinoside | 9.097 | C_27_H_31_O_15_ | [M]+ | 28338-59-2 | 595.1422 | 595.1663 | 4.7 |
| 45 | Cyanidin 3-(6-p-caffeoyl)glucoside | 6.475 | C_30_H_27_O_14_ | [M+H]+ |  | 611.1383 | 611.1401 | 2.9 |
| 46 | Cyanidin 3-(6''-acetylglucoside) | 8.332 | C_23_H_22_O_12_ | [M+H]+ |  | 491.1177 | 490.1111 | 1.4 |
| 47 | Cyanidin 3-O-(6''-glucosyl-2''-xylosylgalactoside) | 5.767 | C_32_H_39_O_20_ | [M+H-H2O]+ | 60029-67-6 | 726.1970 | 743.2035 | 1.3 |
|  | Apigenin and its derivatives |  |  |  |  |  |  |  |
| 48 | Apiin | 6.305 | C_26_H_28_O_14_ | [M+H]+ | 26544-34-3 | 565.1541 | 564.1479 | 1.6 |
| 49 | Apigenin | 6.433 | C_15_H_10_O_5_ | [M+H]+ | 520-36-5 | 271.0598 | 270.0528 | 1.0 |
|  | Naringenin and its derivatives |  |  |  |  |  |  |  |
| 50 | Naringin | 6.305 | C_27_H_32_O_14_ | [M+H-H2O]+ | 10236-47-2 | 563.1699 | 580.1792 | 3.3 |
| 51 | Naringenin | 10.073 | C_15_H_12_O_5_ | [M-H]- | 480-41-1 | 271.0609 | 272.0685 | 0.3 |
| 52 | Chalconaringenin | 12.115 | C_15_H_12_O_5_ | [M-H]- | 5071-40-9 | 271.0609 | 272.0685 | 1.1 |
| 53 | Naringin chalcone | 5.940 | C_27_H_32_O_14_ | [M+H]+ |  | 581.1280 | 580.1792 | 1.1 |
| 54 | Naringenin 7-O-beta-D-glucoside | 8.283 | C_21_H_22_O_10_ | [M+H]+ | 529-55-5 | 435.1308 | 434.1213 | 3.7 |
| 55 | 8-C-Glucosylnaringenin | 7.508 | C_21_H_22_O_10_ | [M+H]+ |  | 435.1284 | 434.1213 | 0.4 |
| 56 | Liquiritin and its derivatives |  |  |  |  |  |  |  |
| 57 | (2S)-Liquiritigenin | 5.768 | C_15_H_12_O_4_ | [M+H]+ | 578-86-9 | 257.0805 | 256.0736 | 1.5 |
| 58 | Isoliquiritin | 10.140 | C_21_H_22_O_9_ | [M-H2O-H]- | 5041-81-6 | 399.1073 | 418.1264 | 1.8 |
| 59 | Genistein | 12.523 | C_15_H_10_O_5_ | [M-H]- | 446-72-0 | 269.0449 | 270.0528 | 1.2 |
| 60 | Genistin | 6.592 | C_21_H_20_O_10_ | [M-H]- | 529-59-9 | 431.0984 | 432.1056 | 0.2 |
| 61 | 6''-Malonylgenistin | 7.088 | C_24_H_22_O_13_ | [M+H-H2O]+ | 51011-05-3 | 501.1006 | 518.1060 | 4.1 |
| 62 | Pelargonidin 3-sophoroside | 7.043 | C_27_H_31_O_15_ | [M]+ | 54542-60-8 | 595.1425 | 595.1663 | 2.6 |
| 63 | peonidin | 13.820 | C_16_H_13_O_6_ | [M-H]- | 134-01-0/ | 300.2622 | 301.2708 | 4.4 |
| 64 | Peonidin-3-glucoside | 7.955 | C_22_H_23_O_11_ | [M+H]+ | 68795-37-9 | 463.1218 | 463.1240 | 2.9 |
| 65 | Peonidin 3-(6''-p-coumarylglucoside) | 7.697 | C_31_H_28_O_13_ | [M+H]+ |  | 609.1583 | 608.1530 | 1.8 |
| 66 | Isovitexin 2''-O-beta-D-glucoside | 7.548 | C_27_H_30_O_15_ | [M+H]+ | 60767-80-8 | 595.1422 | 594.1585 | 3.9 |
| 67 | Luteolin | 9.228 | C_15_H_10_O_6_ | [M+H]+ | 491-70-3 | 287.0548 | 286.0477 | 0.6 |
| 68 | Luteolin 7-glucoside | 6.170 | C_21_H_20_O_11_ | [M-H2O-H]- | 5373-11-5 | 429.0821 | 448.1006 | 0.3 |
| 69 | Isorhamnetin | 10.457 | C_16_H_12_O_7_ | [M-H]- | 480-19-3 | 315.0512 | 316.0583 | 0.6 |
| 70 | Biochanin A | 13.785 | C_16_H_12_O_5_ | [M-H]- | 491-80-5 | 283.1698 | 284.0685 | 1.1 |
| 71 | Rutin | 7.460 | C_27_H_30_O_16_ | [M+H]+ | 153-18-4 | 611.1626 | 610.1534 | 1.6 |
| 72 | Vanillin | 4.987 | C_8_H_8_O_3_ | [M+H]+ | 121-33-5 | 153.0549 | 152.0473 | 1.3 |
|  | **Phenolic acids** |  |  |  |  |  |  |  |
| 1 | Salicylic acid | 5.373 | C_7_H_6_O_3_ | [M-H]- | 69-72-7 | 137.0238 | 138.0317 | 2.7 |
| 2 | 5-Carboxyvanillic acid | 5.590 | C_9_H_8_O_6_ | [M-H]- | 2134-91-0 | 211.0240 | 212.0321 | 3.9 |
| 3 | Gallic acid | 8.552 | C_7_H_6_O_5_ | [M-H]- | 149-91-7 | 169.0144 | 170.0215 | 1.0 |
| 4 | Chlorogenic acid | 6.568 | C_16_H_18_O_9_ | [M+H]+ | 327-97-9 | 355.1013 | 354.0951 | 0.1 |
| 5 | Isochlorogenic acid | 6.427 | C_25_H_24_O_12_ | [M-H]- | 14534-61-3 | 515.1187 | 516.1268 | 1.6 |
|  | benzoic acid and its derivatives |  |  |  |  |  |  |  |
| 6 | 3-Hydroxybenzoic acid | 3.802 | C_7_H_6_O_3_ | [M-H]- | 99-06-9 | 137.0238 | 138.0317 | 4.6 |
| 7 | 4-Hydroxybenzoic acid | 11.445 | C_7_H_6_O_3_ | [M-H]- | 99-96-7 | 137.0239 | 138.0317 | 3.6 |
|  | **Tannins** |  |  |  |  |  |  |  |
| 1 | Ellagic acid | 13.583 | C_14_H_6_O_8_ | [M-H]- | 476-66-4 | 300.9984 | 302.0063 | 2.0 |
| 2 | Procyanidin B2 | 5.495 | C_30_H_26_O_12_ | [M+H]+ | 29106-49-8 | 579.1468 | 578.1424 | 3.5 |

**Supplementary Table S4. Purity of free and bound polyphenol extracts from *R. roxburghii* DGs**

| Polyphenol extracts | | Purity (%) |
| --- | --- | --- |
| Free polyphenol | CS | 46.01 ± 0.17^c^ |
|  | HC | 43.87 ± 0.26^d^ |
| Bound polyphenol | CS | 66.41 ± 0.65^a^ |
|  | HC | 62.80 ± 0.34^b^ |

Two kins of *R. roxburghii* DGs named CS and HC were provided by Changshun Dnansoya *Rosa roxburghii* Farm Co. Ltd. (Guizhou, China) and Guizhou Hongcai Investment Group Co. Ltd. (Guizhou, China), respectively. The purity of free or bound polyphenol extract was expressed as the ratio between the total polyphenol content and the total quality of the extract, expressed as a percentage. Results were expressed as means ± SD, n = 3, and different letters in the same column indicate a significant difference (*p* < 0.05).


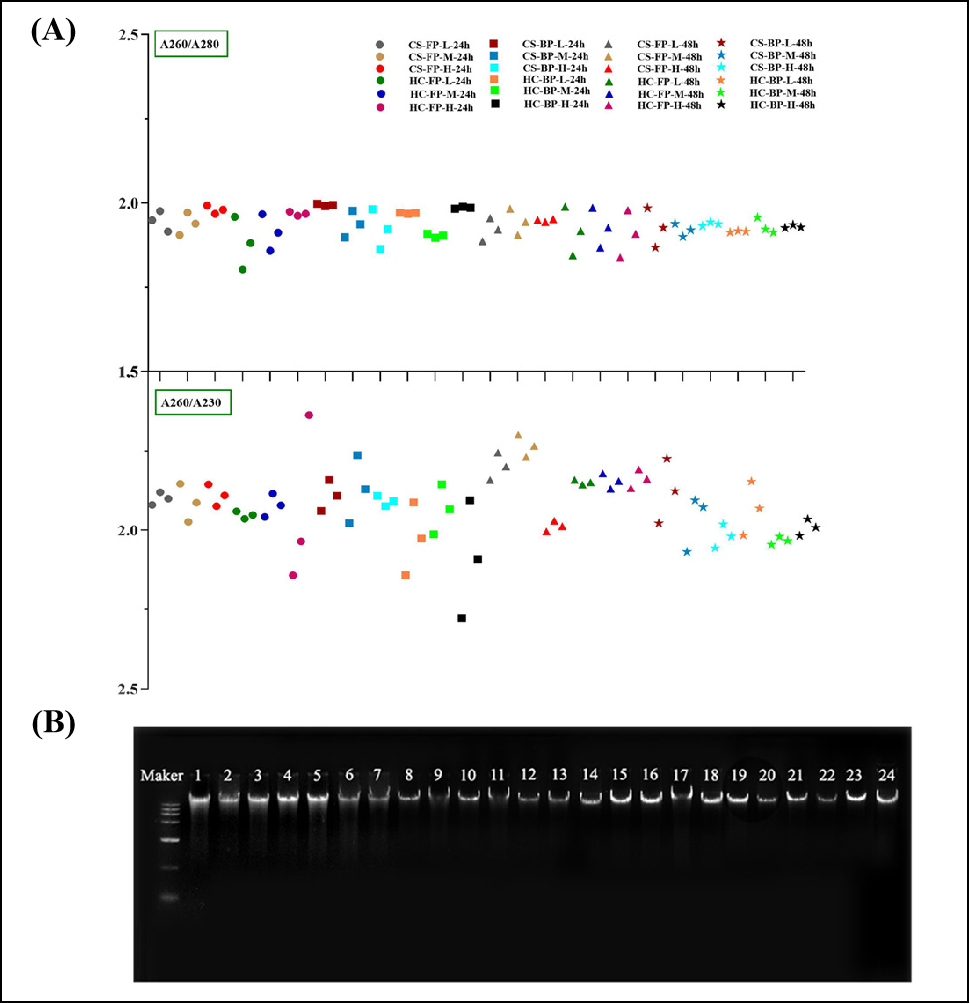


**Supplementary Figure S1. Quality and integrity of the total bacterial genomic DNA:** (A) Values of A260/A280 and A260/A230 of the genomic DNA; (B) Agarose gel electrophoresis. 1-3 represented CS-FP-L-24h, CS-FP-M-24h and CS-FP-H-24h; 4-6 represented CS-BP-L-24h, CS-BP-M-24h and CS-BP-H-24h; 7-9 represented HC-FP-L-24h,HC-FP-M-24h and HC-FP-H-24h; 10-12 represented HC-BP-L-24h, HC-BP-M-24h and HC-BP-H-24h;13-15 represented CS-FP-L-48h, CS-FP-M-48h and CS-FP-H-48h;16-18 represented CS-BP-L-48h, CS-BP-M-48h and CS-FP-H-48h; 19-21 represented HC-FP-L-48h, HC-FP-M-48h and HC-FP-H-48h; 22-24 represented HC-BP-L-48h, HC-BP-M-48h and HC-BP-H-48h. Maker was 15 kb. The free and bound polyphenols from CS (Changshun Dnansoya *Rosa roxburghii* Farm Co. Ltd) were named as CS-FP and CS-BP; the free and bound polyphenols from HC (Guizhou Hongcai Investment Group Co. Ltd.) were named as HC-FP and HC-BP, respectively.

**References:**

Aloisio, I., Santini, C., Biavati, B., Dinelli, G., Cencic, A., Chingwaru, W., Mogna, L., & Di Gioia, D. (2012). Characterization of *Bifidobacterium* spp. strains for the treatment of enteric disorders in newborns. *Applied Microbiology and Biotechnology*, 96(6), 1561-1576. https://doi.org/'10.1007/s00253-012-4138-5.

Gorzelak, M. A., Gill, S. K., Tasnim, N., Ahmadi-Vand, Z., Jay, M., & Gibson, D. L. (2015). Methods for improving human gut microbiome data by reducing variability through sample processing and storage of stool. *PLoS One*, 10(8), e0134802. https://doi.org/'10.1371/journal.pone.0134802.

Kennedy, N. A., Walker, A. W., Berry, S. H., Duncan, S. H., Farquarson, F. M., Louis, P., Thomson, J. M., Consortium, U. I. G., Satsangi, J., Flint, H. J., Parkhill, J., Lees, C. W., & Hold, G. L. (2014). The impact of different DNA extraction kits and laboratories upon the assessment of human gut microbiota composition by 16S rRNA gene sequencing. *PLoS One*, 9(2), e88982. https://doi.org/'10.1371/journal.pone.0088982.

Louis, P., & Flint, H. J. (2009). Diversity, metabolism and microbial ecology of butyrate-producing bacteria from the human large intestine. *FEMS Microbiol Lett*, 294(1), 1-8. https://doi.org/'10.1111/j.1574-6968.2009.01514.x.

Rinttila, T., Kassinen, A., Malinen, E., Krogius, L., & Palva, A. (2004). Development of an extensive set of 16S rDNA-targeted primers for quantification of pathogenic and indigenous bacteria in faecal samples by real-time PCR. *Journal of Applied Microbiology*, 97(6), 1166-1177. https://doi.org/'10.1111/j.1365-2672.2004.02409.x.

Xu, J., Ahren, I. L., Prykhodko, O., Olsson, C., Ahrne, S., & Molin, G. (2013). Intake of blueberry fermented by *Lactobacillus plantarum* affects the gut microbiota of L-NAME treated rats. *Evid Based Complement Alternat Med*, 2013, 809128. https://doi.org/'10.1155/2013/809128.

Xu., N., Tan., G., Wang., H., & Gai., X. (2016). Effect of biochar additions to soil on nitrogen leaching, microbial biomass and bacterial community structure. *European Journal of Soil Biology*, 74, 1-8. https://doi.org/'10.1016/j.ejsobi.2016.02.004.
